# Clinical Profile and Outcomes of Patients With Systemic Lupus Erythematosus

**DOI:** 10.7759/cureus.68541

**Published:** 2024-09-03

**Authors:** Prasad C Bagare, Akshata Borle, Priya Baluni, Gayatri Gajanan Ekbote, Shashikala Sangale

**Affiliations:** 1 Internal Medicine, Dr. DY Patil Medical College, Hospital and Research Centre, Pune, IND; 2 Rheumatology, Deenanath Mangeshkar Hospital and Research Centre, Pune, IND; 3 Internal Medicine, Byramjee Jeejeebhoy Government Medical College and Hospital, Pune, IND

**Keywords:** treatment outcome, disease activity, demography, clinical features, sle (systemic lupus erythematosus), autoimmunity

## Abstract

Background

Systemic lupus erythematosus (SLE) is a complex autoimmune disorder characterized by relapsing-remitting immune system activation, affecting multiple organ systems. Despite significant advances in understanding SLE's pathogenesis, there remains a need for comprehensive clinical profiling at the time of diagnosis to improve early detection and management. This study addresses this gap by providing a detailed analysis of the clinical presentation, disease activity, and patient outcomes using the Systemic Lupus International Collaborating Clinics (SLICC) criteria and Systemic Lupus Erythematosus Disease Activity Index (SLEDAI) index.

Methodology

This cross-sectional observational study included 80 patients diagnosed with SLE using the 2012 SLICC criteria. Patients were recruited from the Rheumatology department and other wards of Byramjee Jeejeebhoy Government Medical College and Sassoon General Hospital, Pune, India. All participants provided informed consent and institutional ethical approval was obtained. Data were collected through detailed clinical history, physical examinations, and standard tests such as chest X-rays, CBC, RFT, LFT, urine microscopy, creatine phosphokinase, ANA, AntiDsDNA, complement consumption, and Coombs' tests, with 2D echocardiography performed as needed. Follow-ups every three months over 1.5 years assessed disease activity using SLEDAI criteria. Patients aged 12 and above who met the SLICC criteria were included and those with other connective tissue disorders were excluded. Associations between clinical symptoms and organ involvement were analyzed using the chi-square test with a p-value of <0.05 considered significant.

Results

The study evaluated 80 patients with SLE, revealing a predominantly female cohort (80%) with a mean age of 29.4 years and a standard deviation of 8.3 years, skewed towards younger age groups. Clinical manifestations were diverse; the most common symptoms were (83.75%), oral ulcers (98.75%), and alopecia (95%). Anemia (66.25%) was the most prevalent abnormality, followed by albuminuria and renal abnormalities. Organ involvement was highest in the renal system (50%) and mucocutaneous features, with lower incidences in cardiac, gastrointestinal, and vascular systems. Gender-specific analyses indicated significant differences in SLE nephritis (p=0.048) and autoimmune hemolytic anemia (p=0.046). Autoantibody profiles showed high positivity for ANA (98.8%) and DsDNA (61.3%). Clinical outcomes demonstrated that 68.8% of patients achieved remission and 16.3% experienced organ damage. The SLEDAI scores significantly improved over time, with substantial reductions from baseline to nine months (p<0.001).

Conclusion

In conclusion, this study provides a detailed examination of SLE, revealing that it predominantly affects young adults and is characterized by diverse manifestations including mucocutaneous symptoms, significant renal involvement, and notable autoantibody profiles. The high prevalence of anti-nucleosome and anti-dsDNA antibodies underscores their diagnostic and prognostic value. Clinically, the findings highlight the necessity for early detection and targeted management of SLE, particularly in addressing renal and mucocutaneous symptoms. Future research should focus on longitudinal studies to track disease progression, explore genetic and environmental influences, and investigate regional variations to enhance treatment strategies and patient outcomes.

## Introduction

Systemic lupus erythematosus (SLE) is a complex systemic autoimmune disease characterized by the immune system's failure to maintain self-tolerance, leading to an immune attack on the host's tissues. This failure can result from the immune system's inability to differentiate between self and non-self or from misinterpreting a self-component as harmful. Autoimmune disorders like SLE represent a significant health burden, affecting 3-9% of the general population [1]. These disorders can be either organ-specific or systemic, with SLE serving as a prime example of the latter, affecting multiple organ systems.

SLE is one of the most extensively researched autoimmune diseases due to its complex pathophysiology, diverse clinical manifestations, and significant impact on patient quality of life. The disease is characterized by the production of autoantibodies that contribute to inflammation and tissue damage across various organ systems. Despite advances in understanding SLE, the exact mechanisms underlying its pathogenesis remain a topic of ongoing research and debate. This includes the role of genetic predisposition, hormonal influences, and environmental triggers in the disease's onset and progression.

The term "lupus erythematosus" was initially coined in the nineteenth century to describe skin lesions, but it wasn't until later that the systemic nature of the disease was recognized [2]. Today, SLE is understood as a chronic, relapsing-remitting condition with a highly variable prognosis. Timely diagnosis is crucial, as delays can lead to irreversible damage to vital organs [3]. Clinicians typically rely on the 2012 Systemic Lupus International Collaborating Clinics (SLICC) criteria for diagnosing SLE, which incorporates both clinical symptoms and laboratory findings, including autoantibody profiles [4,5].

This study aims to fill a research gap by providing a detailed analysis of the clinical presentation and progression of SLE in a specific patient population. It will assess disease activity at the time of diagnosis and over time using the Systemic Lupus Erythematosus Disease Activity Index (SLEDAI) criteria, offering insights into the severity and variability of the disease [6]. Additionally, the study will evaluate patient outcomes to better understand the efficacy of current treatments and the overall prognosis for individuals with SLE.

## Materials and methods

Study design and setting

The study included consecutive patients diagnosed with SLE who visited the rheumatology department or were admitted as inpatients at Byramjee Jeejeebhoy (BJ) Government Medical College and Sassoon General Hospital, a tertiary care hospital in Pune, India. It was a cross-sectional, observational study conducted with an estimated sample size of 60 patients to achieve an accuracy of ±5% and a 95% confidence level. Ethical clearance for the study was obtained from the BJ Government Medical College and Sassoon General Hospital ethics committee (IEC/2023/07/01) before the study commenced.

Selection Criteria

Male or female patients 12 years of age or older who met the eligibility requirements for SLE as stated by the SLICC and provided written informed consent to participate were included in the study. Patients under the age of 12 with connective tissue illnesses other than SLE or mixed connective tissue conditions and those who did not provide written consent or fulfill the SLICC criteria for SLE were excluded from the study. Although the diagnosis of these patients was made after the release of the 2019 American College of Rheumatology/European League Against Rheumatism (ACR/EULAR) criteria, the 2012 SLICC criteria were used for classification. This choice was based on the fact that the study was initiated and the majority of patient diagnoses were confirmed before the 2019 criteria became widely adopted in clinical practice. Consequently, the 2012 SLICC criteria were the prevailing standard at the time of diagnosis for this cohort.

Data sources and variables

The research comprised patients with SLE identified based on the 2012 SLICC Criteria who visited our hospital's medicine outpatient department (OPD) and inpatient department (IPD) departments in addition to inpatients in other departments. We acquired informed permission from each individual. Patients were included regardless of the length of the illness or if they were receiving therapy. From the time the condition first manifested, a thorough history was obtained, and any noteworthy results from systemic and general clinical exams were recorded. Standard investigative reports were compiled for individuals with SLE, including data from direct and indirect Coombs' tests, complete blood count (CBC), renal function tests (RFT), liver function tests (LFT), urine routine microscopy, creatinine phosphokinase (CPK), chest X-ray, antinuclear antibody (ANA), AntiDsDNA and complement consumption reports. The treating physician decided which patients would undergo 2D echocardiography based on the patients' symptoms and the objective findings from clinical, laboratory, and other imaging modalities. Records were kept of unusual reports. All information was recorded on a proforma. Every detail was recorded on a proforma. Over the course of 1.5 years, patients were monitored every three months, and the SLEDAI criterion was used to gauge the extent of illness development.

Statistical analysis

The research population's clinical and demographic features were compiled using descriptive statistics. For continuous data, the mean, median, and standard deviations were computed; for categorical variables, frequencies and percentages were utilized. The Chi-square test was used to examine the relationship between clinical symptoms and organ involvement. P-values below 0.05 were regarded as statistically significant. All statistical analyses were carried out using SPSS software, ensuring precise computations and accurate data interpretation.

## Results

Table 1 describes the study demographics and parameters. The study evaluated 80 patients with SLE, revealing a predominantly female cohort (80%) with a mean age of 29.4 years and a standard deviation of 8.3 years, skewed towards younger age groups. In the age group ≤ 20, there were six male patients (7.5%) and 19 female patients (23.75%), totaling 25 patients or 31.3% of the sample. For the 21-30-year age group, there were six males (7.5%) and 27 females (33.75%), comprising 33 patients or 41.3% of the sample. The 31-40- year age group, comprised three males (3.75%) and 10 females (12.5%), totaling 13 patients or 16.3% of the sample. In the 41-50-year age group, there was one male (1.25%) and six females (7.5%), accounting for seven patients or 8.8% of the sample. In the age group > 50, there were no male patients and two female patients (7.5%), totaling two patients or 2.5% of the sample. Overall, the study included 16 male patients (20%) and 64 female patients (80%), making up a total of 80 patients.

**Table 1:** Study demographics and parameters

Age group	Gender		Percentage (%)	Total
	Male	Female		
≤ 20	6 (7.5%)	19 (23.75%)	31.3	25
21 - 30	6 (7.5%)	27 (33.75%)	41.3	33
31 - 40	3 (3.75%)	10 (12.5%)	16.3	13
41 - 50	1 (1.25%)	6 (7.5%)	8.8	7
> 50	0	2 (7.5%)	2.5	2
Total	16 (20%)	64 (80%)	100	80

Table 2 describes the clinical profile of patients. Rashes were observed in 11 male patients (13.75%) and 56 female patients (70%), making a total of 67 patients, or 83.75% of the sample. Oral ulcers were present in 15 male patients (18.75%) and 64 female patients (80%), making up 79 patients or 98.75% of the sample. Alopecia was observed in 16 males (20%) and 60 females (75%), comprising 79 patients or 95% of the sample. Fever affected 10 male patients (12.5%) and 37 female patients (46.25%), totaling 47 patients or 58.75%. Joint pain was noted in five males (6.25%) and 19 females (23.75%), totaling 24 patients or 30% of the sample. Chest pain was reported in five male patients (6.25%) and 20 female patients (25%), making up 25 patients or 31.25% of the sample. Pedal edema was found in 14 males (17.5%) and 47 females (58.75%), totaling 61 patients or 76.25%. Synovitis was present in eight males (10%) and 29 females (36.25%), totaling 37 patients or 46.25%. Headache affected five male patients (6.25%) and 18 female patients (22.5%), making up 23 patients or 28.75% of the sample. Lastly, abdominal distension was seen in seven males (8.75%) and 30 females (37.5%), totaling 37 patients or 46.25%.

**Table 2:** Clinical profile of patients

Symptoms	Gender		Total	Percentage (%)
	Male	Female		
Rash	11 (13.75%)	56 (70%)	67	83.75
Oral ulcer	15 (18.75%)	64 (80%)	79	98.75
Alopecia	16 (20%)	60 (75%)	79	95.00
Fever	10 (12.5%)	37 (46.25%)	47	58.75
Joint pain	5 (6.25%)	19 (23.75%)	24	30.00
Chest pain	5 (6.25%)	20 (25%)	25	31.25
Pedal edema	14 (17.5%)	47 (58.75%)	61	76.25
Synovitis	8 (10%)	29 (36.25%)	37	46.25
Headache	5 (6.25%)	18 (22.5%)	23	28.75
Abdominal distension	7 (8.75%)	30 (37.5%)	37	46.25

Table 3 summarizes the key laboratory findings among patients with SLE. The most prevalent abnormality was anemia, observed in 66% of patients, followed by leucopenia in 27.5% and thrombocytopenia in 18.75%. Renal function tests showed abnormalities in 40% of patients, while liver function tests, specifically SGOT/SGPT levels, were deranged in 5% of patients. Albuminuria was present in 60% of patients, and renal albumin-to-creatinine ratio (ACR) was abnormal in the same proportion. Furthermore, 38.75% of patients had biopsy-proven lupus nephritis, indicating significant renal involvement in a substantial portion of the study cohort.

**Table 3:** Laboratory findings SGOT: serum glutamic oxaloacetic transaminase; SGPT: serum glutamic pyruvic transaminase

Abnormal laboratory findings	Number of patients	Percentage (%)
Anemia	53	66.25
Leucopenia/ lymphopenia	22	27.5
Thrombocytopenia	15	18.75
Creatinine	32	40
SGOT/SGPT	4	5
Albuminuria	48	60
Albumin/creatinine Ratio	48	60
Renal biopsy	31	38.75

Table 4 presents an overview of organ involvement among patients with SLE. The most prevalent clinical features included oral ulcers (98%), alopecia (95%), and rash (83%), which are components of mucocutaneous involvement. However, when considered as a combined category under mucocutaneous involvement, it was present in 23.80% of patients. Nephritis, affecting 50% of patients, represented the most common renal manifestation. Cardiac involvement was observed in 5% of patients, with pericarditis being the most common manifestation. Pulmonary involvement was noted in 31% of patients, including conditions such as pleural effusion and interstitial lung disease and central nervous system (CNS) involvement in 25%. Gastrointestinal (GIT) manifestations were less common, reported in 3.75% of patients. Vascular involvement and secondary antiphospholipid syndrome (APLA) were among the least observed organ involvements.

**Table 4:** Overall organ involvement GIT: gastrointestinal tract; APLA: antiphospholipid antibody

Organ involvements	Number	Percentage (%)
Mucocutaneous	19	23.80
Hematological	9	11.30
Renal	40	50
Cardiac	4	5
Pulmonary	25	31.35
Neurological	20	25
GIT	3	3.75
Vascular	2	2.50
Secondary APLA syndrome	1	1.25

Table 5 describes the distribution of clinical manifestations and organ involvement in SLE patients by gender. For SLE minor organ involvement, minor organ involvement was classified as any clinical manifestation affecting organs other than the major organs typically considered in SLE, such as the skin or mucous membranes, but not reaching the severity required for classification as major organ involvement. In this study, one male patient (1.25%) and 18 female patients (22.5%) were noted to have minor organ involvement, totaling 19 patients or 23.75% of the sample. Conversely, the absence of minor organ involvement was observed in 15 males (18.75%) and 46 females (57.5%), amounting to 61 patients or 76.25% of the sample. The overall sample consisted of 16 male patients (20%) and 64 female patients (80%) out of 80 patients. The p-value of 0.1 indicates no statistically significant gender difference in minor organ involvement (Table 5).

**Table 5:** Distribution of clinical manifestations and organ involvement in SLE patients by gender ILD: interstitial lung disease; DAH: diffuse alveolar hemorrhage; SLE: systemic lupus erythematosus; CNS: central nervous system A chi-square test was done. P-value < 0.05 was considered to be statistically significant

Category		Count (Male)	Count (Female)	Total	Percentage (%)	p-value
SLE minor organ	Present	1 (1.25%)	18 (22.5%)	19	23.75	0.1
	Absent	15 (18.75%)	46 (57.5%)	61	76.25	
	Total	16 (20%)	64 (80%)	80	100	
SLE nephritis	Present	12 (15%)	28 (35%)	40	50	0.048
	Absent	4 (5%)	36 (45%)	40	50	
	Total	16 (20%)	64 (80%)	80	100	
Cardiorespiratory	Abnormal	1 (1.25%)	6 (7.5%)	7	8.8	0.999
	Normal	15 (18.75%)	58 (72.5%)	73	91.3	
	Total	16 (20%)	64 (80%)	80	100	
Cardiorespiratory abnormality	No cardiorespiratory abnormality	15 (18.75%)	58 (72.5%)	73	91.3	0.239
	Pericardial effusion	0	3 (3.75%)	3	3.8	
	Coronary artery disease	1 (1.25%)	0	1	1.3	
	Pleural effusion	0	2 (2.5%)	2	2.5	
	ILD	0	2 (2.5%)	2	2.5	
	DAH	0	1 (1.25%)	1	1.3	
CNS	Abnormal	4 (5%)	16 (20%)	20	25	0.999
	Normal	12 (15%)	48 (60%)	60	75	
	Total	16 (20%)	64 (80%)	80	100	
CNS sub-group	No CNS Involved	12 (15%)	48 (60%)	60	75	0.599
	Hemiparesis	1 (1.25%)	1 (1.25%)	2	2.5	
	Lupus headache	2 (2.5%)	6 (7.5%)	8	10	
	Seizure	1 (1.25%)	9 (11.25%)	10	12.5	
Autoimmune hemolytic anemia	Present	3 (3.75%)	6 (7.5%)	9	11.3	0.046
	Absent	23 (28.75%)	48 (60%)	71	88.8	
	Total	26 (32.5%)	54 (67.5%)	80	100	

Lupus nephritis was present in 50% of the patients, with 12 males and 28 females affected. The distribution of nephritis classification by gender revealed that 17 patients (42.5%) had unspecified nephritis, with 4 males and 13 females. In class I, there was one female patient (2.5%), while class II had two female patients (5%). Class III included one male and two female patients, totaling three patients or 7.5%. Class IV comprised four males and four females, making up eight patients or 20%. Class V had one male and six female patients, amounting to seven patients or 17.5%. Class VI included two male patients, with no females, totaling two patients or 5%. The p-value of 0.048 suggests a significant gender difference in nephritis prevalence (Table 5).

Cardiorespiratory involvement is defined as abnormalities in heart rate, respiratory rate, blood pressure, or oxygen saturation outside normal ranges (heart rate: 60-100 bpm, respiratory rate: 12-20 breaths/min, blood pressure: 90-120/60-80 mmHg, oxygen saturation: 95-100% on room air), one male (1.25%) and six females (7.5%) were classified as having abnormal cardiorespiratory status, totaling seven patients or 8.8%. Normal cardiorespiratory status was noted in 15 males (18.75%) and 58 females (72.5%), making up 73 patients or 91.3% of the sample. The total count includes 16 male patients (20%) and 64 female patients (80%) out of 80 patients, with a p-value of 0.999. In terms of cardiorespiratory abnormality, 15 males (18.75%) and 58 females (72.5%) had no abnormality, totaling 73 patients or 91.3%. Specific abnormalities included pericardial effusion in three females (3.75%), coronary artery disease in one male (1.25%), pleural effusion in two females (2.5%), ILD in two females (2.5%), and DAH in one female (1.25%). The p-value for cardiorespiratory abnormality is 0.239 (Table 5).

For CNS involvement, 4 males (5%) and 16 females (20%) had abnormalities, totaling 20 patients or 25%. Normal CNS was observed in 12 males (15%) and 48 females (60%), making up 60 patients or 75% of the sample. The overall total includes 16 male patients (20%) and 64 female patients (80%) with a p-value of 0.999. Within the CNS sub-group, 12 males (15%) and 48 females (60%) had no CNS involvement, totaling 60 patients or 75%. Hemiparesis was observed in one male (1.25%) and one female (1.25%), lupus headache in two males (2.5%) and six females (7.5%), and seizure in one male (1.25%) and nine females (11.25%). The p-value for the CNS subgroup is 0.599 (Table 5).

Lastly, for autoimmune hemolytic anemia, three males (3.75%) and six females (7.5%) were affected, totaling nine patients or 11.3%. Autoimmune hemolytic anemia was absent in 23 males (28.75%) and 48 females (60%), making up 71 patients or 88.8%. The total number of patients was 26 males (32.5%) and 54 females (67.5%) out of 80, with a p-value of 0.046.

Table 6 details the autoantibody profile and their associations in SLE patients. ANA was the most prevalent autoantibody, detected in 79 patients, accounting for 98.8% of the study population. DsDNA was the next most common, observed in 49 patients or 61.3% of cases. Anti-Sm antibodies were present in 12 patients, representing 15% of the cohort. Among antiphospholipid antibodies, anti-cardiolipin antibodies were found in five patients (6.25%), anti-β2-glycoprotein I antibodies in three patients (3.75%), and lupus anticoagulant in two patients (2.5%). Secondary antiphospholipid syndrome (APLS) was identified in just one patient, making up 1.25% of the cases. Complement consumption, indicated by low C3 and C4 levels, was observed in 18 patients, which corresponds to 22.5% of the study population.

**Table 6:** Autoantibody profile and their associations ANA: antinuclear antibody; DsDNA: double-stranded DNA; anti-SM: anti-Smith antibody; APLA: antiphospholipid antibodies The presence of APLA does not equate to a diagnosis of APLS.

Autoimmune (antibody)	Number of patients	Percentage (%)
ANA	79	98.8
DsDNA	49	61.3
Anti-Sm	12	15
Anti-cardiolipin antibodies	5	6.25
Anti-β2-glycoprotein I antibodies	3	3.75
Lupus anticoagulant	2	2.5
Secondary antiphospholipid syndrome	1	1.25
Complement consumption (Low C3/C4)	18	22.5

Table 7 shows the clinical outcomes of SLE patients. Among the 80 patients, 13 (16.3%) experienced organ damage, while 55 (68.8%) achieved remission. The clinical outcomes, including organ damage and remission, were assessed using the SLICC criteria, which allowed for a consistent evaluation of disease progression and patient outcomes. There were five patients (6.3%) who had a relapse, and seven patients (8.8%) who unfortunately passed away. The causes of death among these patients were primarily attributed to complications related to SLE, such as severe renal failure, infections due to immunosuppression, and cardiovascular complications.

**Table 7:** Clinical outcomes of SLE patients SLE: systemic lupus erythematosus

Outcome	Number of patients	Percentage (%)
Organ damage	13	16.30
Remission	55	68.80
Relapse	5	6.30
Death	7	8.80
Total	80	100

Table 8 summarizes the progression of the SLEDAI score over time in the study population. At baseline, there were 74 patients, representing 92.5% of the total, with a mean SLEDAI score of 17.01±5.15. At the three-month follow-up, 73 patients (91.25%) were assessed, and the mean SLEDAI score had decreased to 14.28±5.07, with a p-value of < 0.001, indicating a statistically significant reduction. At the six-month follow-up, 73 patients (91.25%) were again assessed, and the mean SLEDAI score further decreased to 7.90±5.44, with a p-value of < 0.001, showing continued significant improvement. Finally, at the nine-month follow-up, 73 patients (91.25%) were evaluated, and the mean SLEDAI score dropped to 5.78±6.24, with a p-value of < 0.001, indicating sustained and significant improvement in disease activity over time.

**Table 8:** SLEDAI score progression over time A p-value < 0.05 was considered significant; A paired t-test was conducted. SLEDAI: Systemic Lupus Erythematosus Disease Activity Index

SLEDAI score	Number of patients	Percentage (%)	SLEDAI	p-value
			Mean±SD	
Baseline	74	92.50	17.01±5.15	
3 months	73	91.25	14.28±5.07	< 0.001
6 months	73	91.25	7.90±5.44	< 0.001
9 months	73	91.25	5.78±6.24	< 0.001

Table 9 presents changes in the SLEDAI scores across multiple follow-up periods. At baseline, the scores were distributed as follows: 0 in the 1-5 score group, six in the 6-10 group, 48 in the 11-19 group, and 26 in the ≥20 group. At the first follow-up, scores shifted with increases observed in some groups: one in the 1-5 score group, 14 in the 6-10 group, and decreases in others: 48 in the 11-19 group, 11 in the ≥20 group. Subsequent follow-ups showed varying trends: significant increases in scores for the 1-5 and 11-19 groups, and decreases in the 6-10 and ≥20 groups, resulting in comparable total scores across all periods, totaling 80 at baseline and 73 at subsequent follow-ups.

**Table 9:** SLEDAI score changes over follow-up periods by score group SLEDAI: Systemic Lupus Erythematosus Disease Activity Index

SLEDAI score group	SLEDAI score			
	Baseline	1st follow-up	2nd follow-up	3rd follow-up
1 - 5	0	1	50	57
6 - 10	6	14	9	4
11 - 19	48	48	10	7
≥ 20	26	11	4	5
Total	80	74	73	73

Table 10 shows the treatment regimens administered to the patients. Hydroxychloroquine (HCQ) and steroids were universally used, with all 80 patients (100%) receiving these treatments. Cyclophosphamide was administered to 31 patients, accounting for 38.75% of the sample. Azathioprine was prescribed to 16 patients, representing 20% of the cohort. Leflunomide or methotrexate was given to seven patients, making up 8.75% of the group, while mycophenolate mofetil (MMF) was used in four patients, accounting for 5% of the total population.

**Table 10:** Treatment

Treatment	Number of patients	Percentage (%)
Hydroxychloroquine (HCQ)	80	100
Steroids	80	100
Cyclophosphamide	31	38.75
Azathioprine	16	20
Leflunomide/methotrexate	7	8.75
Mycophenolate mofetil (MMF)	4	5

## Discussion

SLE is a multifaceted autoimmune condition characterized by diverse clinical manifestations impacting various organ systems. The present study provides insights into the demographic and clinical profile of SLE, consistent with global and regional research. Key findings include a predominant occurrence among young adults, with mucocutaneous symptoms such as rashes and oral ulcers being the most prevalent. Renal involvement, particularly lupus nephritis, was prominent, alongside significant hematological and cardiac manifestations. Autoantibody profiles, notably anti-nucleosome and anti-dsDNA antibodies, exhibited associations with specific clinical features, highlighting their diagnostic and prognostic implications.

In the present study, the median age of participants was 24 years, with the majority between the ages of 21-30, consistent with findings from Kosaraju et al. [7], Agrawal et al. [8], and other global studies such as Mahmoudi et al. [9] and Rees et al. [10]. The female-to-male ratio was approximately 4:1, aligning with ratios reported internationally ranging from 8-15:1 [11]. Studies in India by Malaviya et al. [12], Binoy et al. [13], and Kosaraju et al. [7] have reported varying ratios (8:1, 19:1, and 15:1 respectively), potentially influenced by cohort sizes and diagnostic practices.

Mucocutaneous involvement, including rashes, oral ulcers, hair loss, and photosensitivity, was prominent, and consistent with global and local studies [7,14]. Specifically, oral ulcers and alopecia were highly prevalent (98%), similar to findings by Saigal et al. [14]. Arthritis prevalence varied, with higher rates reported elsewhere [12,13].

Kidney involvement, predominantly lupus nephritis, affected 50% of the cohort, with biopsy-proven nephritis in 38.75%, primarily diffuse proliferative nephritis as per International Society of Nephrology/Renal Pathology Society (ISN/RPS) classification, comparable to findings by Kosaraju et al. [7]. Hematological manifestations were observed in 66.25% of patients, aligning with other studies [8,9].

Cardiac involvement, identified via 2D echocardiography in 5% of patients, highlighted pericarditis as the most common finding, similar to previous reports [12-14]. Lung involvement was noted in 31.35% of patients, including pleural effusion and interstitial lung disease, consistent with findings by Malaviya et al. [12] and Binoy et al. [13].

Neuropsychiatric symptoms were present in 25% of patients, with seizures being the most common manifestation (12.5%), comparable to previous reports [6,11-13]. Gastrointestinal involvement was minimal (3.75%), differing from higher rates reported elsewhere [15]. Ocular involvement was rare (13.1%), predominantly with sicca symptoms, similar to global findings.

The study also assessed autoantibody profiles, with a high prevalence of anti-nucleosome antibodies (98.8%) and associations with nephritis, fever, arthritis, and AIHA, consistent with previous research [16-18]. Anti-dsDNA antibodies were found in 61.3% of patients, significantly associated with nephritis, malar rash, oral ulceration, and arthritis, in line with existing literature [7,19,20].

Anti-Sm antibodies were present in 15% of patients, showing associations with nephritis, malar rash, photosensitivity, and fever, similar to findings by Alba et al. [19] and Tang et al. [21]. Overall, the study contributes to understanding SLE in the population, highlighting similarities and unique aspects compared to global and regional studies.

Limitations of the study

This study has several limitations that should be acknowledged. Firstly, the cohort included both newly diagnosed and existing SLE patients. Specifically, 20 patients were newly diagnosed at enrollment, while the remaining 40 had a history of SLE. New clinical features emerged in eight patients (13.3%) after enrollment, reflecting the progressive nature of the disease. Although efforts were made to update clinical details during follow-up visits, the detection of new features during the study period may have influenced the overall findings. Additionally, variability in the sensitivity and specificity of ELISA and immunodot blot assays could affect the detection of low-titer antibodies, which may fluctuate and become undetectable over time. The inherently episodic nature of SLE, characterized by periods of relapse and remission, further complicates the interpretation of longitudinal data, as antibody titers and disease manifestations can change in response to disease activity and treatment regimens.

## Conclusions

In conclusion, this study offers significant insights into the demographic and clinical profiles of SLE within our cohort, aligning with global patterns in age distribution and gender ratios. The study highlights the prevalence of diverse manifestations including mucocutaneous, renal, hematological, cardiac, neuropsychiatric, and autoimmune symptoms, emphasizing the complex and multifaceted nature of SLE. These findings not only enhance our understanding of SLE's varied presentations but also suggest the need for a more individualized approach in clinical management. Clinicians should consider incorporating regular screening for specific manifestations such as lupus nephritis and neuropsychiatric symptoms, given their high prevalence and impact on patient outcomes. Additionally, the study underscores the importance of utilizing autoantibody profiles for more accurate diagnosis and prognosis. Future research should focus on longitudinal studies to explore the progression of these manifestations and the effectiveness of targeted interventions, ultimately guiding the development of more personalized treatment strategies for SLE.
